# Socioeconomic factors associated with choice of delivery place among mothers: a population-based cross-sectional study in Guinea-Bissau

**DOI:** 10.1136/bmjgh-2018-001341

**Published:** 2019-04-03

**Authors:** Sanni Yaya, Ghose Bishwajit, Nathali Gunawardena

**Affiliations:** 1 School of International Development and Global Studies, University of Ottawa, Ottawa, Ontario, Canada; 2 Faculty of Science, University of Ottawa, Ottawa, Ontario, Canada

**Keywords:** facility delivery, global health, antenatal care, guinea bissau, maternal healthcare, institutional delivery, women of reproductive age

## Abstract

**Background:**

Maternal death outcome remains high in Guinea-Bissau. Delivery-related complications and maternal mortality could be prevented by increasing women’s access to skilled pregnancy care. Socioeconomic status (SES) is often associated with low health service utilisation in low/middle-income countries. In Guinea-Bissau, little is known on the relationship between SES and use of health facility for delivery. In this study, we examined the association between women’s choice of health facility delivery with their SES.

**Methods:**

Current data from Multiple Indicator Cluster Survey conducted in Guinea-Bissau, 2014 were used in this study. The place of delivery (home or health facility) was the outcome variable of interest using 7532 women of reproductive age (15–49 years). Respondents’ characteristics were described by summary statistics, while multivariable logistic regression model was used to examine the association of demographic and socioeconomic characteristics on facility-based delivery. Adjusted ORs, 95% CIs and p values were computed to identify significant determinants.

**Results:**

Results show that in proportion of women delivering at home was higher than of delivery at a health facility. Overall percentage of women who delivered at health facility was 39.8%, with the rate being substantially higher among urban (67.8%) compared with their rural (30.2%) counterparts. Percentage of delivering at home was highest in Oio region (23.8%) and that of delivery at a health facility was highest in the Sector Autónomo de Bissau region (18.7%). In the multivariable analysis, women in urban areas compared those who had no education, those who had primary and secondary/higher level of education were 2.2 and 3.3 times more likely to deliver at a health facility. The odds of facility were also highest among the women from the richest households, 5.3 and 5 times among urban and rural women, respectively.

**Conclusion:**

Based on these findings, the study concludes that the percentage of health facility delivery is low in Guinea-Bissau, which can be promoted through scaling up women’s SES. The findings could guide healthcare policy-makers to address the issue of unskilled delivery services and increase the use of facility-based delivery particularly among the disadvantaged women.

**Key questions**

**What is already known?**

- Institutional delivery plays a vital role in reducing maternal and child mortality.
- A large proportion of women in Africa are deprived from basic maternal healthcare services.

**What are the new findings?**

- More than three-fifths of the women in Guinea-Bissau do not deliver at health facilities.
- Women from lower socioeconomic status are significantly less likely to use health facility delivery services.

**What do the new findings imply?**

- Improving women’s socioeconomic position can promote the utilisation of health facility delivery services.
- Health policy-makers should make sure that women’s inability to pay does n’ot barrier their access to essential maternal health services.

## Background

Choice of healthcare provider in sub-Saharan Africa is often limited. Only 3% of the world’s healthcare workforce is found in the region despite the area having 24% of the global disease burden.^1^ Despite progress in the area of expanding the range of healthcare services in the region,^2^ as well as retaining healthcare professionals,^3^ people living in sub-Saharan Africa, especially those in rural areas, continue to have a limited choice of healthcare services. The poorest members of society in low/middle-income countries, especially in resource-limited settings, tend to use medical services far less than others.^4^

In low/middle-income countries, the impact of an individual’s socioeconomic status (SES) on their utilisation of healthcare services has been extensively documented. In Burkina Faso, individuals from disadvantaged communities seek healthcare services considerably less than others.^5^ This same trend is seen in Ethiopia where poorer households use healthcare services less often than those from wealthier households.^6^ Both direct barriers, such as charges and fees,^7^ as well as indirect barriers such as transport costs, limit the use of health services by those of low SES.^8^

In Guinea-Bissau, a small West African country with a population of 1.8 million people, poverty is a major issue with the GDP per capita being just US$352 per person. The country ranked 10th among 192 countries on under-five child mortality with 203 per 1000 in 2004.^9^ There are likely multiple factors resulting in high child mortality in rural areas of Guinea-Bissau, one of which includes low income.^10^ In Guinea-Bissau, 80.4% of the population (1201 thousand people) are multidimensionally poor while an additional 10.5% live near multidimensional poverty (156 thousand people).^11^

Overall, identifying determinants of facility-based delivery is a major step to improve maternal health outcomes, based on high maternal mortality rate in low-income and middle-income countries.^12^ However, in Guinea-Bissau, there is paucity of data from studies showing predictors of the choice of facility-based delivery. Understanding the relationship between women’s SES and choice of healthcare facility can assist with the development of interventions and policy changes for key populations in order to improve health outcomes for women and children. This study aims to examine the association between women’s choice of health facility delivery with their SES in Guinea-Bissau with the research question, to extent are women’s SES associated with choice of delivery place? Data for this study were collected from the Multiple Indicator Cluster Survey (MICS) conducted in Guinea-Bissau in 2014.

## Methods

### Data source

Data for this survey were collected from the fifth round of the MICS-5 conducted in Guinea-Bissau among 7532 women of reproductive age (15–49 years). MICS is a multinational household survey initiative conducted by Unicef and designed to fill data gaps for monitoring the situation of children and women. Unicef, through the MICSs, has transformed the data landscape in the past 20 years. MICS findings are used to for policy decisions and programme interventions related to the situation of children and women around the world. MICS consists of five questionnaires including: Household Questionnaire, Individual Questionnaire for Women, Questionnaire for Children under Five, Questionnaire for Men and Questionnaire for Children aged 5–17. The MICSs provide information on items such as education level, wealth status and use of healthcare services.

The main objectives of the survey were: to provide up-to-date information for assessing the health status of children and women (including men) and to monitor progress towards the Millennium Development Goals and assist in targeted interventions thereby. The survey was conducted in 2014 by the Ministry of Economy and Finance through the Direcção Geral do Plano/Instituto Nacional de Estatística, within the scope of the Global MICS Programme. The United Nations Children’s Fund (Unicef) provided technical and financial support for conducting the survey. Additional financial and logistical contributions were provided by the United Nations Development Programme, the United Nations Population Fund, PLAN Guinea-Bissau and the International Partnership for Human Development. MICS-5 was nationwide sample survey encompassing all nine regions in the country: Tombali, Quinara, Oio, Biombo, Bolama/Bijagós, Bafatá, Gabú, Cacheu and Sector Autónomo de Bissau (SAB). Details on sampling procedure are available on the final report.^13^

### Variables

The main outcome variable was place of delivery. This was categorised as home delivery and facility delivery. Main explanatory variable was SES of women, which was proxy by their educational and wealth status. Several covariates were included in the analysis as potential confounders: age, marital status, region, religion, access to television (TV), radio and internet, frequency of antenatal care (ANC) attendance.

### Data analysis

At first, we checked the data for multicollinearity and ran distribution tests for assess normality. Any outliers were removed. Then the dataset was weighted using the sample weight variable provided in the dataset. Data analysis included descriptive statistics to present the basic characteristics of the participants. Charts were created to visually illustrate the variation in the proportion of facility delivery across the study regions. χ^2^ bivariate tests were performed to select the potential predictors for the multivariable analyses (results were not shown in the analysis). Following that, binary regression analysis was performed to measure the ORs of the association between health facility delivery and the explanatory variables while adjusting for potential confounders. As the outcome variable was dichotomous, we used binary logistic regression model that is an appropriate method for normally distributed data. The level of significance was set at 5%.

We conducted the analyses using publicly available data from demographic health surveys.

### Patient and public involvement

Patients and public were not involved in the design and conduct of this research.

## Results

### Descriptive statistics

Table 1 shows that mean age of the sample women were 31.48 years (SD 4.48). Most of the women were in the 25–29 years (20.2%) age group, were currently married (75.0%), belonged to Islam faith (48.5%), from Norte province (32.8%). Almost half of the women reported listening to radio every day, while 3% reported never using any. Rate of TV watching was less common as 8.5% watched every day, and 17.2% did not watch at all. Only 2.8% of the women reported ever using internet. Proportion of ANC attendance was 11.7%. More than half (55.7%) of the women had no formal education. More than a quarter (27.3%) were living in the poorest households, and only 11.5% in the richest households. The table also shows that majority of the women were of rural origin (67.9%), and compared with rural women, urban women were had higher percentage of accessing radio, TV, internet. Overall proportion of facility delivery was 39.8%. Urban women were less likely than rural women to attend at least four ANC visits, while rural women were less likely to deliver at a health facility compared with their urban counterparts.

**Table 1:** Basic characteristics of the sample population, MICS 2014

Variables	Total		Urban		Rural	
	N=7532	%	N=2418	32.1%	N=5114	67.9%
Age (mean/SD)	31.48/8.48		31.43/8.33		31.51/8.54	
15–19	475	6.3	132	5.5	343	6.7
20–24	1400	18.6	458	18.9	942	18.4
25–29	1525	20.2	514	21.3	1011	19.8
30–34	1427	18.9	468	19.4	959	18.8
35–39	1136	15.1	356	14.7	780	15.3
40–44	903	12.0	269	11.1	634	12.4
45–49	666	8.8	221	9.1	445	8.7
Marital status						
Currently married/in union	5651	75.0	1494	61.8	4157	81.3
Formerly married/in union	636	8.4	297	12.3	339	6.6
Never married/in union	1245	16.5	627	25.9	618	12.1
Religion						
Christian	1787	23.7	935	38.7	852	16.7
Islam	3653	48.5	1185	49.0	2468	48.3
Other	2092	27.8	298	12.3	1794	35.1
Province						
Norte	2471	32.8	405	16.7	2066	40.4
Leste	1768	23.5	438	18.1	1330	26.0
Sul	2164	28.7	446	18.4	1718	33.6
SAB	1129	15.0	1129	46.7		
Radio						
Everyday	3689	49.0	1395	57.7	2294	44.9
Few times/week	2217	29.4	626	25.9	1591	31.1
<Once/week	1402	18.6	354	14.6	1048	20.5
Never	224	3.0	43	1.8	181	3.5
TV						
Everyday	637	8.5	465	19.2	172	3.4
Few times/week	2020	26.8	899	37.2	1121	21.9
<Once/week	3576	47.5	838	34.7	2738	53.5
Never	1299	17.2	216	8.9	1083	21.2
Ever used internet						
Yes	53	2.8	41	6.9	12	0.9
No	1822	97.2	549	93.1	1273	99.1
Received ANC						
Yes	880	11.7	178	7.4	702	13.7
No	6652	88.3	2240	92.6	4412	86.3
Educational attainment						
Nil	4196	55.7	780	32.3	3416	66.8
Primary	2192	29.1	766	31.7	1426	27.9
Secondary/higher	1144	15.2	872	36.1	272	5.3
Wealth status						
Poorest	2058	27.3	102	4.2	1956	38.2
Second	1718	22.8	211	8.7	1507	29.5
Middle	1629	21.6	444	18.4	1185	23.2
Fourth	1261	16.7	858	35.5	403	7.9
Richest	866	11.5	803	33.2	63	1.2
Place of delivery						
Home	1924	60.2	264	32.2	1660	69.8
Health facility	1272	39.8	555	67.8	717	30.2

ANC, antenatal care; MICS, Multiple Indicator Cluster Survey; SAB, Sector Autónomo de Bissau.

**Figure 1:**
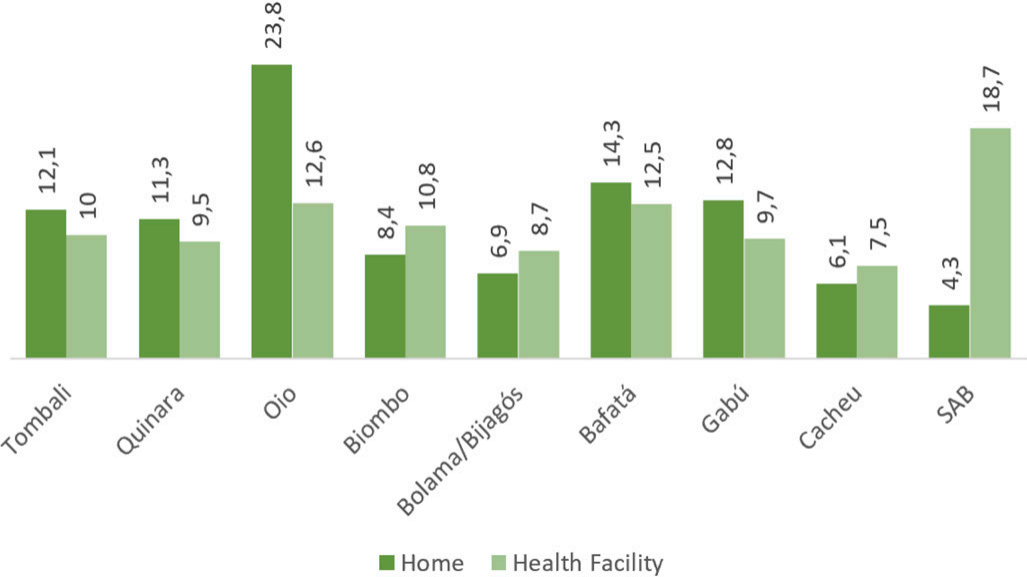
Regional distribution of home and facility delivery (%). SAB, Sector Autónomo de Bissau.

Figure 1 describes the percentage distribution of home and facility delivery across the regions. It shows that in five out of nine the regions, the proportion of delivering at home was higher than of delivery at a health facility. Percentage of delivering at home was highest in Oio region (23.8%) and that of delivery at a health facility was highest in the SAB region (18.7%).

**Figure 2:**
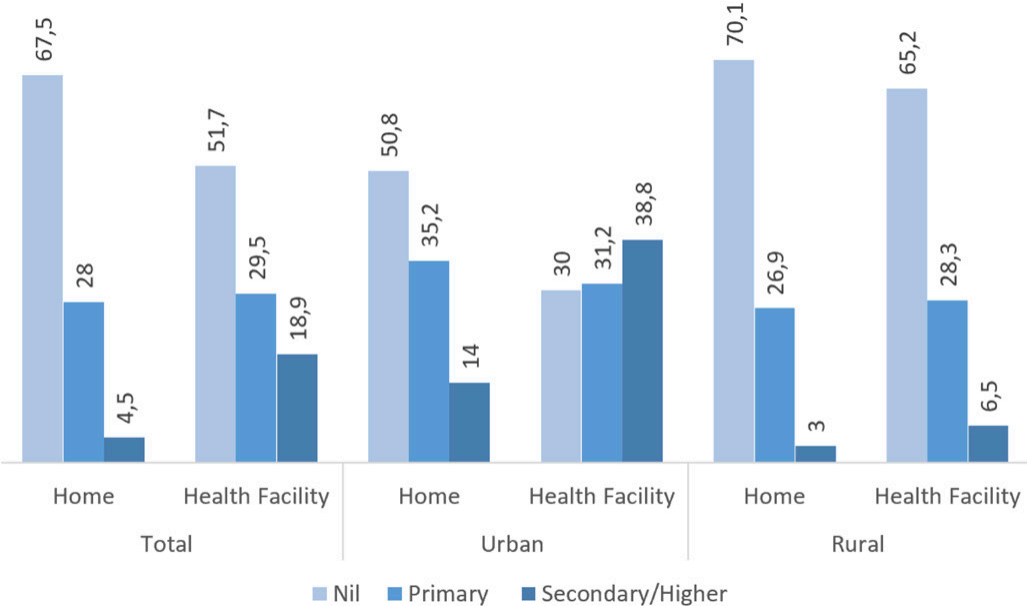
Distribution of home and facility delivery across the educational level (%).

Figure 2 illustrates the percentage distribution of home and facility delivery across the three educational groups. It appears that both in urban and rural areas, home was preferred place of delivery among those who had no education. In the urban areas, those who delivered at a health facility were more likely to have primary or secondary/higher level education.

**Figure 3:**
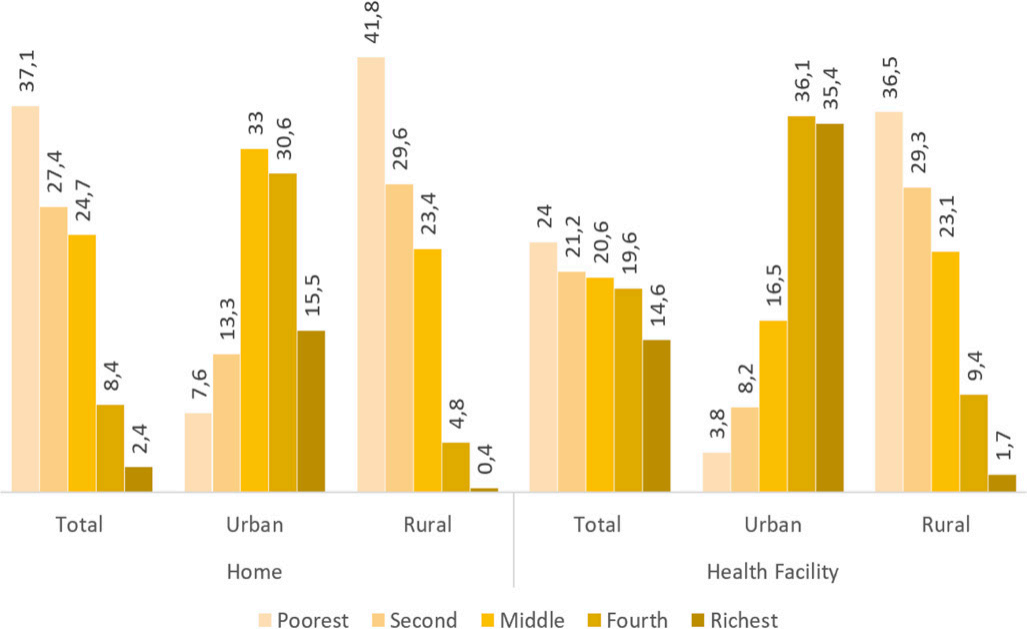
Distribution of home and facility delivery across the wealth quintile (%).

Figure 3 shows the percentage distribution of home and facility delivery across the wealth quintiles.

From the figure, it is clear that those who delivered at home were more likely to be from lower wealth status and less likely to be from higher wealth status households. Conversely, those who delivered at a health facility were more likely to be from higher wealth status and less likely to be from lower wealth status households.

Results of multivariable analysis (see table 2) show that higher educational level and wealth status were associated with higher odds of delivering at health facilities. In the urban areas, compared those who had no education, those who had primary and secondary/higher level of education were 2.2 and 3.3 times more likely to deliver at a health facility. The odds of facility were also highest among the women from the richest households, 5.3 and 5 times among urban and rural women, respectively.

**Table 2:** Multivariate association between women preference for facility deliver with educational and wealth status, MICS 2014

	Urban			Rural		
	AOR	95% CI upper	95% CI lower	AOR	95% CI upper	95% CI lower
Educational attainment						
Nil	Ref			Ref		
Primary	2.269	1.806	2.850	0.753	0.578	1.081
Secondary/higher	3.337	2.607	4.272	0.849	0.644	1.120
Wealth status						
Poorest	Ref			Ref		
Poorer	1.848	1.102	3.101	2.307	1.765	3.017
Middle	2.426	1.641	3.586	2.625	1.998	3.450
Richer	1.977	1.505	2.597	2.605	1.974	3.438
Richest	5.347	4.172	6.853	5.006	3.565	7.031

N.B. Regression analyses were adjusted for age, marital status, religion, province, radio, TV, ever used internet and receiving ANC.

ANC, antenatal care; AOR, adjusted OR; MICS, Multiple Indicator Cluster Survey.

## Discussion

By analysing data from the information gathered by the MICS conducted in Guinea-Bissau in 2014, our study came to several findings about the relationship between women’s SES and their choice of healthcare facility. The study found that women with higher educational attainment and from higher wealth status households were more likely to deliver at healthcare facilities while their less educated and low wealth status counterparts were more likely to deliver at home.

Most of the women surveyed were in the 25–29 years age group, were currently married and belonged to Islam faith, from Norte province. The majority of the women were of rural origin, and compared with rural women, urban women were had higher percentage of accessing radio, TV, internet. Urban women were less likely than rural women to attend at least four ANC visits, while rural women were less likely to deliver at a health facility. Another study conducted in Guinea-Bissau also had similar findings reporting that 75% of women give birth at home in rural areas.^14^

In urban areas, women with primary, secondary or higher level education were found to be more likely to deliver at a healthcare facility. Seventy per cent of women delivering in healthcare facilities in urban areas were those who had primary, secondary or higher level education. In both urban and rural areas, women with no education were more likely to deliver at home with 50.8% of home deliveries in urban areas and 70.1% of home deliveries in rural areas being by women of no education. Interestingly, many secondary or higher level educated women in rural areas were found to not be delivering at healthcare facilities compared with their urban counterparts. Compared with the 70% of women with primary, secondary or higher level education delivering in healthcare facilities in urban areas, only 34.8% of primary, secondary or higher level educated women delivered in healthcare facilities in rural settings. In a Kenyan study, long distances from health facilities rather than economic or cultural factors were found to be the main reason why women chose to deliver at home.^15^ Long distances to health facilities are usually more prevalent in rural areas, providing a possible explanation to our finding that women with higher levels of education tend to deliver at home in rural settings. It was also found that individuals from a lower wealth status were much more likely to deliver at home while those with a higher wealth status were more likely to deliver at a healthcare facility. 64.5% of home births were by women of the poorest and second poorest wealth status while in healthcare facilities, only 45.2% of deliveries were made by women of the poorest and second poorest wealth status. This contrasts women of the richest and second richest wealth status of whom only 10.8% deliver at home while 34.3% deliver at a healthcare facility.

The multivariable analysis also displayed that higher educational level and wealth status were associated with higher odds of delivering at health facilities. In the urban areas, those who had primary and secondary/higher level of education were 2.2 and 3.3 times more likely to deliver at a health facility than those with no education. The odds of delivering at a health facility were also highest among the women from the richest households, 5.3 and 5 times among urban and rural women, respectively. Our findings reflect past studies. Other studies have found educational attainment of mothers to be one of the most important factors associated with utilisation of health facilities for delivery.^16–25^ The finding that wealthier women are more likely to deliver in healthcare facilities more than their poorer counterparts has also been documented in other studies.^19 26–30^

In response to the high level of home births and lack of health facility use among mothers of low SES in Guinea-Bissau, services at healthcare facilities need to be improved and there needs to be an increase in training of birth attendants for at-home deliveries.^31^ Due to the fact that health facility use remains low despite the facilities existing and being available to women,^32^ the focus should be on improving home births and making them safer as a short-term solution. Perinatal and neonatal deaths can be reduced by training birth attendants.^33^ Further research is needed in determining factors influencing women’s utilisation of healthcare facilities as current studies have not been able determine why utilisation of health facilities remains low in low-income countries.^32^

### Strengths and limitations

The major strength of this study is the use of nationally representative dataset. Based on the large data, generalisation of research findings is possible. However, a drawback is recall bias in the study. More so, this study used secondary data. MICS focused on demographic and socioeconomic variables in their interviews, other factors such as cultural norms, accessibility challenges which are capable of influencing facility-based delivery were not captured in the survey instrument.

## Conclusion

Through this study we were able to determine that women’s SES is associated with their choice of healthcare facility in Guinea-Bissau. The study found that women with higher educational attainment and from higher wealth status households were more likely to deliver at healthcare facilities while their less educated and low wealth status counterparts were more likely to deliver at home. Measures should be taken to determine factors that determine women’s decisions in order to improve the quality of healthcare facilities and maternal satisfaction thereby improving the desire for facility births.

## References

[1] World Health Organization The World Health report 2008: Primary health care – now more than ever, 2008 Available: http://www.who.int/whr/2008/en/index.html

[2] International Finance Corporation (2006) Africa Health Care Report & IFC Strategy, 2006 Available: http://www.ifc.org/ifcext/media.nsf/AttachmentsByTitle/SM09_AfricaHealthCare_IssueBrief/$FILE/SM09_AfricaHealthCare_IssueBrief.pdf

[3] Kumar P Providing the providers - remedying Africa's shortage of health care workers. N Engl J Med 2007;356:2564–7. 10.1056/NEJMp078091 17582065

[4] Dunlop S, Coyte PC, McIsaac W Socio-economic status and the utilisation of physicians' services: results from the Canadian national population health survey. Soc Sci Med 2000;51:123–33. 10.1016/S0277-9536(99)00424-4 10817475

[5] Develay A, Sauerborn R, Diesfeld HJ Utilization of health care in an African urban area: results from a household Survey in Ouagadougou, Burkina-Faso. Soc Sci Med 1996;43:1611–9. 10.1016/S0277-9536(96)00061-5 8961405

[6] Reniers G, Tesfai R Health services utilization during terminal illness in Addis Ababa, Ethiopia. Health Policy Plann 2009;4:1–8. 10.1093/heapol/czp015 PMC3202702 19372240

[7] Haddad S, Fournier P, Quality FP Quality, cost and utilization of health services in developing countries. A longitudinal study in Zaïre. Soc Sci Med 1995;40:743–53. 10.1016/0277-9536(94)00134-F 7747209

[8] Kroeger A, Anthropological KA Anthropological and socio-medical health Care research in developing countries. Soc Sci Med 1983;17:147–61. 6836349 10.1016/0277-9536(83)90248-4

[9] UNICEF The state of the world's children 2006: excluded and invisible. Available: http://www.unicef.org/sowc06/fullreport/full_report.php

[10] Mann V, Fazzio I, King R, et al The EPICS trial: enabling parents to increase child survival through the introduction of community-based health interventions in rural guinea Bissau. BMC Public Health 2009;9 10.1186/1471-2458-9-279 PMC2736942 19650919

[11] United Nations Development Programme Human development report 2016. Guinea-Bissau, 2016.

[12] World Healthhealth Statistics Maternal mortality ratio. 24, 2010.

[13] Ministério da Economia e Finanças, Direcção Geral do Plano/Instituto Nacional de Estatística (INE) Inquérito aos Indicadores Múltiplos (MICS5) 2014, Relatório final. Bissau Guiné-Bissau: Ministério da Economia e Finanças e Direcção Geral do Plano/ Instituto Nacional de Estatística (INE), 2014.

[14] Hoj L, da Silva D, Hedegaard K, et al Factors associated with maternal mortality in rural Guinea-Bissau. A longitudinal population-based study. BJOG: An Internal Journal of Obs Gyn 2002;109:792–9. 10.1111/j.1471-0528.2002.01259.x 12135216

[15] Moindi RO, Ngari MM, Nyambati VCS, et al Why mothers still deliver at home: understanding factors associated with home deliveries and cultural practices in rural coastal Kenya, a cross-section study. BMC Public Health 2016;16 10.1186/s12889-016-2780-z PMC4738797 26842657

[16] Shiferaw S, Spigt M, Godefrooij M, et al Why do women prefer home births in Ethiopia? BMC Pregnancy Childbirth 2013;13 10.1186/1471-2393-13-5 PMC3562506 23324550

[17] Kebede B, Gebeyehu A, Andargie G Use of previous maternal health services has a limited role in reattendance for skilled institutional delivery: cross-sectional survey in Northwest Ethiopia. Int J Womens Health 2013;5:79–85. 10.2147/IJWH.S40335 23459063 PMC3583437

[18] Varma D, Khan ME, Hazra A Increasing institutional delivery and access to emergency obstetric care services in rural Uttar Pradesh. The Journal of Family Welfare 2010;56:23–30.

[19] Kitui J, Lewis S, Davey G Factors influencing place of delivery for women in Kenya: an analysis of the Kenya demographic and Health survey, 2008/2009. BMC Pregnancy Childbirth 2013;13 10.1186/1471-2393-13-40 PMC3585789 23414104

[20] Odo DB, Shifti DM Institutional delivery service utilization and associated factors among child bearing age women in Goba Woreda, Ethiopia. JGO 2014;2:63–70. 10.11648/j.jgo.20140204.14

[21] Kamal SMM Factors affecting utilization of skilled maternity care services among married adolescents in Bangladesh. Asian Popul Stud 2009;5:153–70. 10.1080/17441730902992075

[22] Gazali WA, Muktar F, Gana MM Barriers to utilization of maternal health care facilities among pregnant and non-pregnant women of child bearing age in maiduguri metropolitan Council (MMC) and jere lgas of borno state. CJT Med 2012;6:1–21.

[23] Tura G Antenatal care services utilization and associated factors in Metekel zone, Northwest Ethiopia. Ethiop J Health Sci 2009;19:111–9.

[24] Awoke W, Muhammed J, Abeje G Institutional delivery service utilization in Woldia, Ethiopia. Science Journal of Public Health 2013;1:18–23. 10.11648/j.sjph.20130101.13

[25] Bhattacharyya S, Srivastava A, Roy R, et al Factors influencing women's preference for health facility deliveries in Jharkhand state, India: a cross sectional analysis. BMC Pregnancy Childbirth 2016;16 10.1186/s12884-016-0839-6 PMC4782569 26951787

[26] Agha S, Carton TW Determinants of institutional delivery in rural Jhang, Pakistan. Int J Equity Health 2011;10 10.1186/1475-9276-10-31 PMC3159141 21801437

[27] Anyait A, Mukanga D, Oundo GB, et al Predictors for health facility delivery in Busia district of Uganda: a cross sectional study. BMC Pregnancy Childbirth 2012;12 10.1186/1471-2393-12-132 PMC3514288 23167791

[28] Koenig MA, Jamil K, Streatfield PK, et al Maternal health and care-seeking behavior in Bangladesh: findings from a national survey. Int Fam Plan Perspect 2007;33:075–82. 10.1363/3307507 17588851

[29] Zere E, Oluwole D, Kirigia JM, et al Inequities in skilled attendance at birth in Namibia: a decomposition analysis. BMC Pregnancy Childbirth 2011;11 10.1186/1471-2393-11-34 PMC3118957 21569585

[30] Wagle RR, Sabroe S, Nielsen BB Socioeconomic and physical distance to the maternity hospital as predictors for place of delivery: an observation study from Nepal. BMC Pregnancy Childbirth 2004;4 10.1186/1471-2393-4-8 PMC425583 15154970

[31] UNICEF Accelerating child survival and development: a results-based approach in high under-five mortality areas: final report to CIDA. New York City, 2005.

[32] Kerber KJ, de Graft-Johnson JE, Bhutta ZA, et al Continuum of care for maternal, newborn, and child health: from slogan to service delivery. The Lancet 2007;370:1358–69. 10.1016/S0140-6736(07)61578-5 17933651

[33] Lewin S, Lavis JN, Oxman AD, et al Supporting the delivery of cost-effective interventions in primary health-care systems in low-income and middle-income countries: an overview of systematic reviews. The Lancet 2008;372:928–39. 10.1016/S0140-6736(08)61403-8 18790316

